# Exposure to mild blast forces induces neuropathological effects, neurophysiological deficits and biochemical changes

**DOI:** 10.1186/s13041-018-0408-1

**Published:** 2018-11-09

**Authors:** Adan Hernandez, Chunfeng Tan, Florian Plattner, Aric F. Logsdon, Karine Pozo, Mohammad A. Yousuf, Tanvir Singh, Ryan C. Turner, Brandon P. Luke-Wold, Jason D. Huber, Charles L. Rosen, James A. Bibb

**Affiliations:** 1 Department of Psychiatry, University of Texas Southwestern Medical Center, Dallas, TX 75390 USA; 2 Center for Translational Neurodegeneration Research, University of Texas Southwestern Medical Center, Dallas, TX 75390 USA; 3 Department of Neurosurgery, West Virginia University School of Medicine, Morgantown, WV 26506-9183 USA; 4 Department of Basic Pharmaceutical Sciences, West Virginia University School of Medicine, Morgantown, WV 26506-9530 USA; 5 Departments of Surgery, Neurobiology, and Neurology, The University of Alabama at Birmingham Medical Center, 1720 2nd Ave S, THT 1052, Birmingham, AL 35294 USA

**Keywords:** Blast-induced traumatic brain injury, Neuroinflammation, Microvascular damage, Axonal swelling, Short-term plasticity, Calpain, p25

## Abstract

Direct or indirect exposure to an explosion can induce traumatic brain injury (TBI) of various severity levels. Primary TBI from blast exposure is commonly characterized by internal injuries, such as vascular damage, neuronal injury, and contusion, without external injuries. Current animal models of blast-induced TBI (bTBI) have helped to understand the deleterious effects of moderate to severe blast forces. However, the neurological effects of mild blast forces remain poorly characterized. Here, we investigated the effects caused by mild blast forces combining neuropathological, histological, biochemical and neurophysiological analysis. For this purpose, we employed a rodent blast TBI model with blast forces below the level that causes macroscopic neuropathological changes. We found that mild blast forces induced neuroinflammation in cerebral cortex, striatum and hippocampus. Moreover, mild blast triggered microvascular damage and axonal injury. Furthermore, mild blast caused deficits in hippocampal short-term plasticity and synaptic excitability, but no impairments in long-term potentiation. Finally, mild blast exposure induced proteolytic cleavage of spectrin and the cyclin-dependent kinase 5 activator, p35 in hippocampus. Together, these findings show that mild blast forces can cause aberrant neurological changes that critically impact neuronal functions. These results are consistent with the idea that mild blast forces may induce subclinical pathophysiological changes that may contribute to neurological and psychiatric disorders.

## Introduction

Blast-induced traumatic brain injury (bTBI) results from direct or indirect exposure to an explosive event as may occur in domestic or industrial accidents, terrorist attacks, or in military conflicts [1, 2]. Primary bTBI is induced by a blast overpressure wave that penetrates the skull and causes physical damage to neurons, glia and vasculature. Primary bTBI is normally characterized by internal injuries that are difficult to detect and assess for severity. Parameters affecting the severity of primary bTBI include proximity to explosion focus, the force of the explosion, as well as the duration and characteristics of the explosion. While strong blasts may cause severe and acute brain injury or death, exposure to mild blast forces may result in delayed or subclinical neuropathological changes. bTBI is comorbid with an increased incidence of neuropsychiatric disorders and long-term physical, cognitive, behavioral, and emotional changes [3]. Furthermore, bTBI has been suggested to contribute to the pathogenesis of neurodegenerative disorders [4, 5].

Over the past decade various animal models using moderate to severe blast forces have been developed to study the pathophysiological effects of bTBI, [4, 6–10]. These studies report characteristic neuropathological changes, including neuronal injury, neuroinflammation, hematomas, or contusion in rodent models of bTBI. Moreover, induction of common biochemical and molecular mechanisms associated with neuronal injury have been reported [4, 6, 11, 12]. Together, these studies revealed that primary bTBI induced by stronger blasts negatively affects a variety of cerebral structures and neuronal functions. While these more profound effects demonstrate the ability to model moderate to severe bTBI, the physiological impact of mild blast forces remains poorly defined. It is hypothesized that milder blast forces, still may induce neuropathophysiological changes, however a better understanding of the effect of mild bTBI on neuronal functions is needed.

In order to investigate the effects of mild blast forces on neuropathological, histological, biochemical and neurophysiological outcomes in rodents, we used a scaled, bench-top blast tube set-up [6]. We found that mild blast forces do not induce macroscopic neuropathological changes, including tissue damage, hemorrhage, hematoma and contusion. In fact, mild blast induced microvascular damage, axonal injury and neuroinflammation in various brain regions, including cortex, striatum and hippocampus. Consistent with these neuropathological changes, evaluation of hippocampal synaptic plasticity revealed deficits in short-term plasticity and synaptic excitability after mild blast exposure, but no impairment of hippocampal long-term potentiation (LTP). Finally, brains exposed to mild blast forces exhibited biochemical changes, including proteolytic cleavage of spectrin and formation of the aberrant cyclin-dependent kinase 5 (Cdk5) activator, p25, which have been implicated with neuronal injury and excitotoxicity.

## Methods

### Antibodies and materials

The following antibodies were used, including the phospho-specific neurofilament antibody, SMI-31 (Covance Research Products Inc. Cat # SMI-31, RRID: AB_2314901), glial fibrillary acidic protein (GFAP) antibody (Millipore Cat # AB5804, RRID: AB_2109645), the ionized Ca^2+^-binding adapter molecule (Iba1) antibody (Wako Cat # 019–19741, RRID: AB_839504), the spectrin (α-Fodrin) antibody (Enzo Life Sciences Cat # BML-FG6090–0500, RRID: AB_11179351), the GAPDH antibody (Sigma-Aldrich Cat # G8795, RRID:AB_1078991), the p35 (C-19) antibody (Santa Cruz Biotechnology Cat # sc-820, RRID: AB_632137), Goat anti-mouse IgG (Thermo Fisher Scientific Cat # 31432, RRID: AB_228302) and goat anti-rabbit IgG peroxidase conjugated secondary antibodies (Thermo Fisher Scientific Cat # 31462, RRID:AB_228338). All materials were obtained from Sigma-Aldrich unless stated otherwise.

### Animals

All procedures involving animals were approved by the Institutional Animal Care and Use Committees (IACUC) of West Virginia University and UT Southwestern Medical Center, and were performed according to the principles of the *Guide for the Care and Use of Laboratory Animals*. Male Sprague-Dawley rats were acquired from Hilltop Lab Animals (Hilltop Lab Animals, Inc., Scottsdale, PA, RRID: RGD_734476). At the time of blast exposure the rats were 12 weeks old and weighed ~ 300–350 g. Animals were housed single-caged with a 12 h light/dark cycle and access to food and water ad libitum*.* Prior to experimental use animals were acclimated for 1 week. The study involved the following numbers of animals: neuropathological/histological analysis (18 rats), neurophysiological analysis (17 rats) and biochemical analysis (26 rats). No animals have been excluded from the analyses.

### Blast exposure protocol

**Fig. 1:**
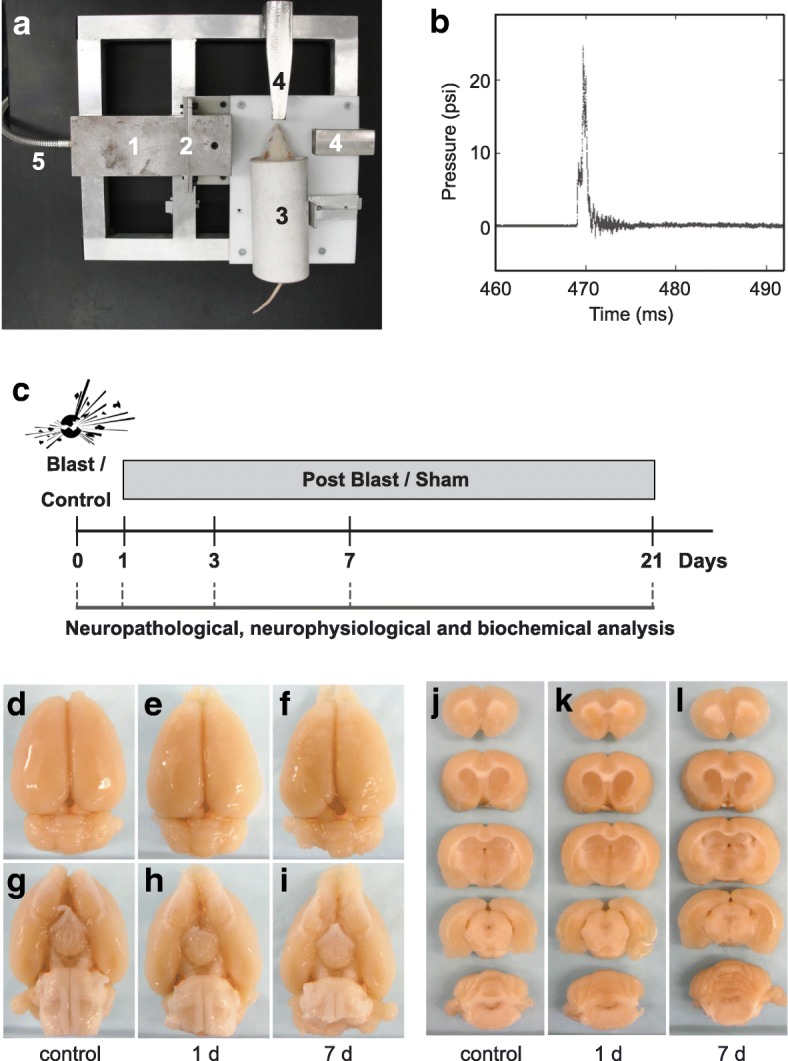
Absence of macroscopic tissue damage after mild bTBI. **a** Image of compressed gas-driven blast tube set-up. *1*) Blast tube (diameter: 7.2 cm) *2*) Membrane (0.076 mm thickness) *3*) rat holding tube *4*) pressure sensors *5*) connection to nitrogen gas tank. **b** Temporal pressure force plot of blast overpressure wave. **c** Experimental timeline. **d**-**i** Absence of macroscopic tissue damage at 1 and 7 day(s) post-bTBI as compared to control. Dorsal view of brain (**d**-**f**); ventral view of brain (**g**-**i**). **j**-**l** No macroscopic tissue damage at 1 and 7 day(s) post-bTBI compared to control as tested in anterior-posterior, 4 mm coronal rat brain sections from controls (**j**), 1 day (**k**) and 7 days (**l**) post-bTBI (*n* = 3/number of animals)

Prior to blast exposure, animals were anesthetized with 4% isoflurane (Henry Schein, Cat # NDC 11695–6776-2). The blast was delivered to the left side of the head with the animal’s body oriented perpendicular to the compressed gas-driven blast tube (length 15 cm; diameter 7.2 cm), and with the peripheral organs protected by a polyvinyl chloride pipe shield (Fig. 1a). The head was placed 12.5 cm from the blast origin. The animals were exposed to a mild blast (0.076 mm membrane; incident peak overpressure of ~ 15 psi.), which was established to cause neuropathophysiological changes, but no external wounds and mortality in previous work [6]. To reduce distress and pain animals are under isoflurane anesthesia during exposure to the mild blast. Immediately following blast exposure, animals were returned to a holding cage equipped with a heating blanket to maintain body temperature at 37 °C. A rectal thermometer was used to monitor body temperature. Once basic reflexes were restored, animals were returned to their home cage and monitored over the next 24 h for adverse reactions. Neuropathological, neurophysiological and biochemical analyses were conducted in rats that had been subjected to one single mild blast overpressure wave and were subsequently sacrificed at 1, 2, 3, 7 or 21 day(s) post-bTBI (see Experimental timeline (Fig. 1c)). Control subjects were anesthetized and placed in proximity to the blast set-up, but were not subjected to blast forces.

### Study design

Animals were randomly assigned to an experimental group (i.e. various post-bTBI delay or control). Neuropathological, neurophysiological and biochemical analyses were performed with the researchers blind to treatment condition. Experimental sample sizes have been based on numbers established in previous experiments (e.g. [13, 14]). No sample size calculation, or power analysis has been performed prior to data collection.

### Neuropathological analysis

The neuropathological analysis was conducted in a sham-treated control group and animals at 1, 3, 7 or 21 day(s) post-blast (*n* = 3 for each time point). Animals were euthanized by CO_2_ asphyxiation and perfused transcardially with ice-cold 0.9% saline followed by 10% formalin for a total of 10 min. The brains were dissected and placed in fresh 10% formalin for 72 h. Brains were block-sectioned into 5 coronal slabs, paraffin-embedded, and serially sectioned at 5 μm at Bregma level of 2.20, 1.00, − 2.80, − 7.30 and − 11.30 mm. Standard protocols were utilized for staining with hematoxylin and eosin (H&E; Leica, Cat # 3801570) [15] and Fluoro-Jade B (FJB, Millipore Corporation, Cat # AG310-30MG) [16]. For the immunohistochemical analysis, paraffin-embedded sections were labeled with SMI-31 to detect phosphorylated neurofilaments (1:500; Covance, Emeryville, CA), glial fibrillary acidic protein (GFAP) to detect astrocyte activation (1:1200; Chemicon) and ionized Ca^2+^-binding adapter molecule (Iba1) to detect microglial activation (1:1000; Wako) and visualized by immunoperoxidase method [17]. Briefly, 5 μm sections were deparaffinized, subjected to microwave antigen retrieval (citrate buffer, pH 6.0; BioGenex, Cat # HK086-9 K), permeabilized with 0.3% (vol/vol) Triton, quenched free of endogenous peroxidases, and blocked with a cocktail of normal goat serums (2.5% (vol/vol) each) prior to overnight incubation of primary antibodies at 4 °C. Bound primary antibodies were detected by sequential incubation with biotinylated secondary antisera and streptavidin-peroxidase, diaminobenzidine chromagen was used to detect immunoperoxidase signal (Vector; anti-mouse IgG kit, Cat # MP-7602, anti-rabbit kit, Cat # MP-7601). Quantification of GFAP-positive astrocytes was conducted using semi-stereology and the optical fractionator technique as previously described [6]. GFAP-positive astrocytes were quantitated in brain regions, including cortex (GFAP-positive cells counted in squares of 100 × 100 μm; *n* = 53–77 squares), striatum (*n* = 32–53 squares) and hippocampus (*n* = 19–34 squares). Semi-quantitative analysis of the immunohistochemical signal of the activated microglia marker, Iba1, and phospho-specific anti-neurofilament antibody, SMI-31 was performed using the open source image processing package FIJI (http://www.fiji.sc). Images were captured on an epifluorescence microscope (Nikon) in TIFF format. Following standardized color deconvolution and thresholding of images, the signal was quantified on 2–3 slides from 3 individual rats for each treatment group. Iba1 was quantified in brain regions, including cortex (% area of signal; *n* = 12–17 regions of interest), striatum (*n* = 11–15 regions of interest) and hippocampus (*n* = 8–12 regions of interest).

### Neurophysiological analysis

Neurophysiological studies were conducted in rats at 1, 3, 7 or 21 day(s) post-bTBI and controls (*n* = 6). Following rapid decapitation and dissection, brains were placed in ice-cold artificial cerebrospinal fluid (ACSF; 75 mM sucrose, 87 mM NaCl, 2.5 mM KCl, 1.25 mM NaH_2_PO_4_, 25 mM NaHCO_3_, 7 mM MgCl_2_, 0.5 mM CaCl_2_ and 10 mM glucose), and transverse hippocampal slices (350 μm) were prepared using a vibratome (Leica Microsystems Inc., VT1000S). Slices were recovered in oxygenated Krebs’ buffer (125 mM NaCl, 2.5 mM KCl, 1.25 mM NaH_2_PO_4_, 25 mM NaHCO_3_, 1.1 mM MgCl_2_, 2 mM CaCl_2_ and 25 mM glucose) at 30 °C for 30 min after slicing. Subsequently slices were moved into oxygenated Krebs’ buffer at room temperature (22–25 °C) before recordings. Slices for recordings were transferred into a perfusion chamber on the upright microscope stage (Axioscop 2, Carl Zeiss, Inc). The perfusion bath was maintained at 30 °C during the recordings (TC-324B Automatic Temperature Controller, Warner Instruments Corporation). A Multiclamp 700A amplifier with a Digidata 1322 and pClamp 10 software (Axon, Molecular devices, LLC) was used for electrophysiological recordings and data acquisition. Field excitatory postsynaptic potentials (fEPSP) from CA1 were evoked by square current pulses (0.1 ms) at 0.033 Hz with a bipolar stimulation electrode (FHC, Bowdoinham, ME) placed at the Schaffer collaterals (~ 250–300 μm) from the recording electrode. Results were obtained using a stimulus intensity to induce 50% of the maximal fEPSP slope and the same intensity was used to explore the paired pulse ratio (PPR) at different intervals. The stimulation intensity was established through the input-output curve, the maximal stimulation was considered when a population spike appeared in the fEPSP. A stable baseline was recorded for at least 15 min prior to high frequency stimulation (HFS, 4 trains, 100 Hz, 1 s duration, separated by 20 s). Post-tetanic potentiation (PTP) was analyzed by taking the average of the slopes from the traces recorded during the first 2 min after HFS. LTP was assessed for at least 60 min after HFS. The time-course showing baselines and LTP is expressed as a percent of change from the baseline fEPSP slope. The PPR values were calculated by dividing the second fEPSP slope by the first fEPSP slope (fEPSP2/fEPSP1). For the input-output curve recordings were normalized for each slice, assigning the maximal fiber volley amplitude (obtained with maximal stimulation) to the value of 1.0, and the plots were derived for the respective fEPSP slopes. All recordings were performed in the absence of any drug treatment and only 1 or 2 slices were recorded from each individual rat. Data were analyzed with Clampfit 10 software (Axon, Molecular devices, LLC). Prism 6 (GraphPad Software, Inc.) was used to make graphs and statistical analysis.

### Quantitative immunoblot analysis

Brain dissection, tissue removal, lysate preparation, and immunoblotting were performed as previously described [18, 19]. Rats for immunoblotting were sacrificed at 1, 3, 7 and 21 day(s) post-bTBI. Tissue lysates from hippocampal dissections were prepared using protease- and phosphatase inhibitors (Roche Diagnostics GmbH, Cat # 05 892 791 001) and equal amounts of protein were run on 7% acrylamide gels or 10–20% gradient acrylamide gels. The blots were probed with primary antibodies raised against spectrin (1:1000, Enzo Life Sciences), p35/25 (1:500, Santa Cruz Biotechnology) and GAPDH (1:5000, Sigma). Signal was detected with HRP-conjugated secondary antibodies (1:1000–10000, Thermo Fisher Scientific) and enhanced chemiluminescence (Thermo Scientific, Cat # 34580). For quantitative immunoblot analysis, immunoreactivity signals were captured by autoradiography, scanned and quantified using ImageJ software (NIH, Bethesda, MD). The band intensities were normalized to signals from GAPDH.

### Statistical analysis

Data are presented as mean ± SEM. Student’s *t*-test or one-way ANOVA were performed to analyze datasets using GraphPad Prism 6 (GraphPad Software Inc., San Diego, CA, USA) unless stated otherwise. A *p*-value < 0.05 was considered statistically significant for all data analyzed. *p < 0.05, **p < 0.01 and ***p < 0.001. For all experiments the whole set of data was analyzed and no data points were excluded. No specific assessment of the normality of data was carried out and no specific test for outliers was conducted, as no data points were excluded.

## Results

### Mild blast forces induce neuroinflammation, but no overt neuropathological effects

Previously, we developed a scaled, bench-top compressed gas-driven blast tube for rodents for the induction of bTBI and characterized the relationship between force intensity and injury severity with regard to general neuropathology using this blast set-up (see Fig. 1a) [6]. These studies defined an incident peak overpressure of approximately 15 psi as a sub-threshold blast force that does not induce overt neuropathological effects, such as tissue damage, hematoma, hemorrhage or contusion. These findings were consistent with those reported in comparable bTBI studies [4, 11, 20, 21].

Here, we first defined the temporal characteristics and the force profile of the blast overpressure wave produced by our bench-top set-up (Fig. 1b). We found that membrane rupture (thickness 0.076 mm) in this system generates a single overpressure pulse lasting a few milliseconds that models the blast waves generated at the start of an explosion. Next, we investigated whether exposure to a single pulse of overpressure in our set-up induced macroscopic neuropathological effects in adult male rats. Gross examination of rat brains at 1, 3, 7 and 21 day(s) following blast exposure revealed no macroscopic evidence of contusion, necrosis, hematoma, hemorrhage, or focal tissue damage of bTBI brains (Fig. 1d-l; data shown for 1 and 7 day(s) post-bTBI, vs. control). In line with our previous observations [6], this result confirms that the blast forces used here to induce bTBI are below the threshold for induction of gross neuropathological effects or damage to the dura.

Previous studies showed that exposure to higher intensity blast forces induce deficits in learning and memory as well as motor performance (e.g. [22]). These functions are dependent upon the hippocampus, cerebral cortex, and striatum. The hippocampus is also highly susceptible to oxidative stress and neuroinflammation, which are acutely increased following TBI [2, 23]. Furthermore, the cerebral cortex is amongst the most susceptible brain areas for neuropathological effects, including neuroinflammation and diffuse axonal injury induced by moderate to strong blast forces [24]. Therefore, we evaluated the ability of mild blast forces to induce subtle neuropathological effects in these brain regions.

**Fig. 2:**
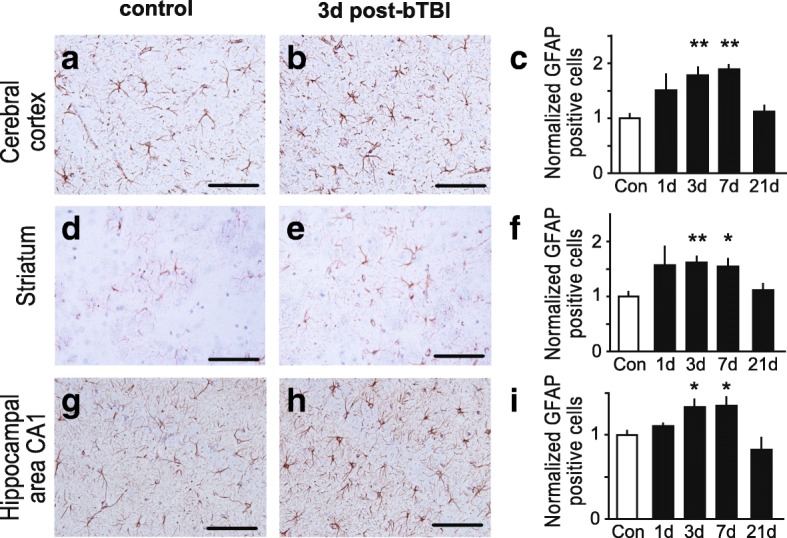
Astrogliosis induction after mild bTBI. **a**-**i** Mild blast forces caused increases in reactive astrocytes throughout the brain, including the brain regions: cerebral cortex (**a**-**c**), striatum (**d**-**f**) and hippocampal area CA1 (**g**-**i**). Representative images of brain regions stained for GFAP, a marker for reactive astrocytes, are shown for controls (**a**, **d** and **g**) and rats at 3 days post-bTBI (**b**, **e** and **h**). Quantifications of GFAP-positive cells expressed as normalized mean cell number per 0.01 mm^2^ are shown for corresponding brain regions for controls and rats at 1, 3, 7 and 21 day(s) post-bTBI (**c**, **f** and **i**). GFAP-positive cells were counted in *n* = 19–77 squares of 100 × 100 μm on slides from 3 individual rats for each treatment group (6–32 squares/rat). All scale bars indicate 50 μm. All data are presented as mean ± SEM; *p < 0.05, **p < 0.01; ANOVA with Bonferroni post hoc

**Fig. 3:**
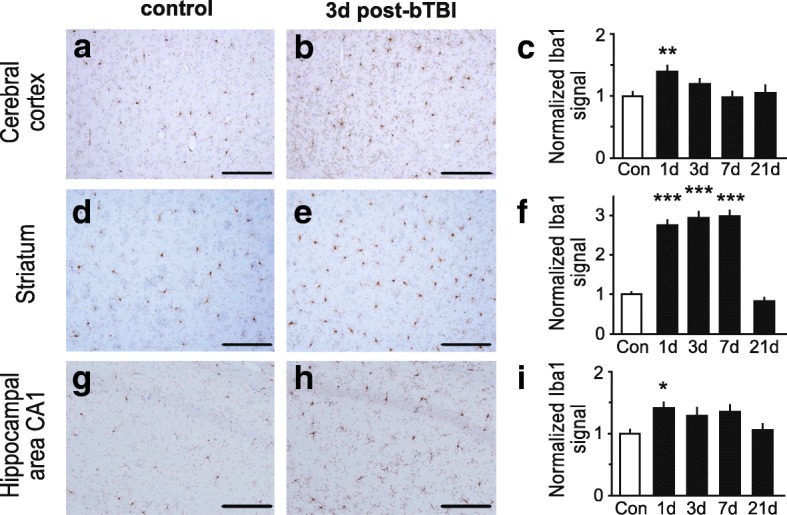
Induction of activated microglia after mild bTBI. **a**-**i** Rats subjected to mild bTBI showed increased levels of activated microglia throughout the brain, including the brain regions: cerebral cortex (**a**-**c**), striatum (**d**-**f**) and hippocampal area CA1 (**g**-**i**). Representative images of brain regions stained for the ionized Ca^2+^-binding adaptor molecule 1 (Iba1), a marker of activated microglia, are shown for controls (**a**, **d** and **g**) and rats at 3 days post-bTBI (**b**, **e** and **h**). Bar graph of the normalized signal of Iba1 for corresponding brain regions for controls and rats at 1, 3, 7 and 21 day(s) post-bTBI is shown (**c**, **f** and **i**). Signal was quantitated on *n* = 6–9 slides from 3 individual rats for each treatment group (2–3 slides/rat). All scale bars indicate 100 μm. All data are presented as mean ± SEM; *p < 0.05, **p < 0.01, ***p < 0.001; ANOVA with Bonferroni post hoc

First, we analyzed the effects of mild bTBI on markers of neuroinflammation immunohistologically in the hippocampus, cerebral cortex, and striatum. As observed in previous studies that used higher intensity bTBI protocols, we found that mild blast induced neuroinflammation in cerebral cortex, striatum and hippocampus, as evidenced by both astrogliosis (Fig. 2) and microglial activation (Fig. 3). The number of reactive astrocytes detected by immunohistochemical staining for GFAP increased throughout the brain, including the cerebral cortex (Fig. 2a-c), striatum (Fig. 2d-f) and hippocampus (Fig. 2g-i) following bTBI. Astrogliosis was evident at 1, 3 and 7 day(s) post-bTBI, but had receded by 21 days to levels comparable to controls (Fig. 2c, f, i). The levels of reactive astrocytes in the ipsilateral brain hemisphere (side facing the blast tube) and the contralateral hemisphere were comparable (data not shown). Brainstem and cerebellum exhibited low levels of astrogliosis (data not shown). In addition, semi-quantitative analysis of Iba 1 immunohistochemical signal showed that levels of activated microglia were increased in the cerebral cortex (Fig. 3a-c), striatum (Fig. 3d-f) and hippocampus (Fig. 3g-i) post-bTBI as compared to non-treated controls. Significant increases in microglia activation could be appreciated at 1, 3 and 7 days post-bTBI in striatum and on day 1 post-bTBI in cortex and hippocampus. Together, these findings show that the mild bTBI protocol used here induces a robust neuroinflammatory response, without causing gross neuropathological effects, such as tissue damage, hemorrhage, or contusion.

### Mild blast forces induce microvascular damage

**Fig. 4:**
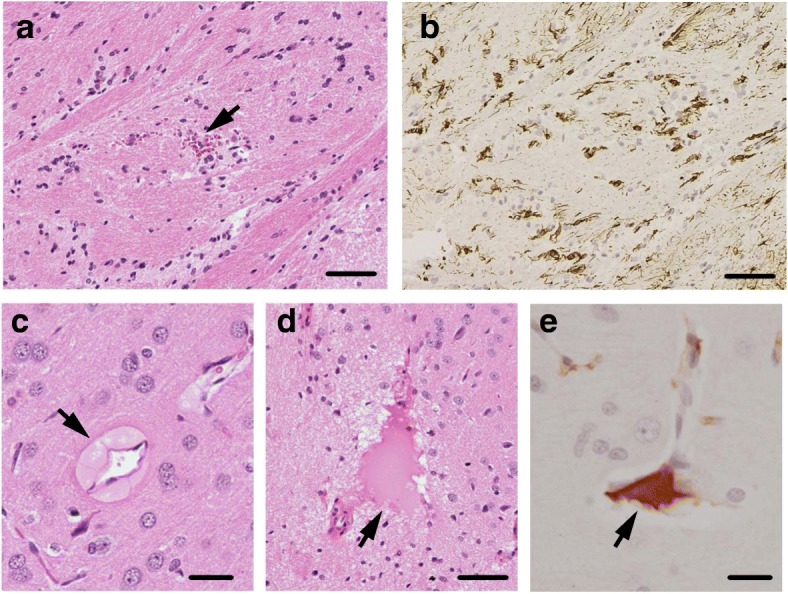
Induction of microvascular damage after mild bTBI. **a** and **b** Microvascular damage in rat brain 3 days post-bTBI as indicated by small hemorrhagic focus (arrowhead) in the corpus callosum (**a**), in conjunction with reactive astrogliosis in the same area (**b**). **c** Extravasation of blood plasma in a venule-like microvessel in the deep layer of the cerebral cortex at 3 days post-bTBI are indicated by an arrowhead. **d** Extravasation of blood plasma in an arteriole-like microvessel in striatum at 3 days post-bTBI. **e** Immunoreactivity for rat immunoglobulin G (IgG) was detected in the same area as the extravasation of blood plasma in (**d**). Representative microscope pictures of brain regions stained with H&E (**a**, **c** and **d**), anti-GFAP antibody (**b**) and anti-rat IgG antibody (**e**) are shown. Analysis included *n* = 3 rats for each treatment group. Scale bars: 50 μm (**a**, **b** and **d**); 20 μm (**c** and **e**)

The presence of a neuroinflammation in absence of macroscopic neuropathological effects, suggests that some microscopic tissue damage may have occurred. Indeed, a detailed microscopic examination of the bTBI brains revealed notable, but sparse alterations to the microvasculature in bTBI brains. Some of the microvascular damage observed included small hemorrhagic foci, which showed the presence of intraparenchymal red blood cells (Fig. 4a) and robust reactive astrogliosis (Fig. 4b) in the striatum of post-bTBI brains. Furthermore, the extravasation of blood plasma was observed in a small number of venule-like (Fig. 4c) and arteriole-like (Fig. 4d) microvessels of the cerebral cortex and striatum post-bTBI. The presence of blood plasma extravasation was confirmed by staining for rat immunoglobulin G (IgG; Fig. 4e). Together, these results show that mild bTBI induced microvascular damage, but this did not lead to hematoma or hemorrhage. Our previous results revealed that higher blast forces correlate with increased damage to the blood vessels, including hematoma and hemorrhage [6]. Our mild blast results may be subtle, but could prove significant, as there is a growing perception that damage to the microvasculature may be an important contributor to the etiology of bTBI [25] and other neurological disorders.

### The effect of mild blast forces on hippocampus

**Fig. 5:**
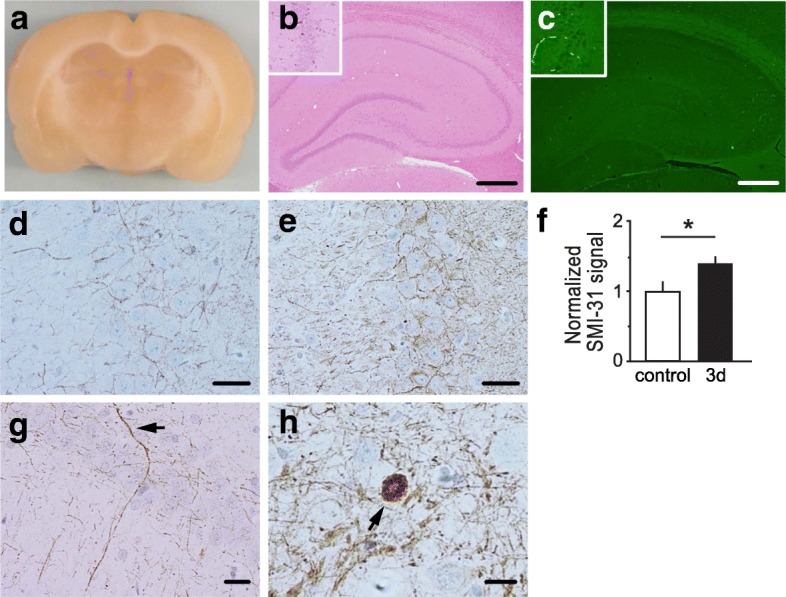
Mild bTBI caused axonal injury in the hippocampus. Mild blast exposure did not result in macroscopic damage, but induced microscopic pathological effects, such as axonal damage and neuroinflammation in the hippocampus. **a** Absence of macroscopic hippocampal tissue damage at 7 days post-bTBI as tested in anterior-posterior 4 mm coronal rat brain sections. **b** Absence of overt neuronal injury in hippocampus at 7 days post-bTBI as assessed with H&E staining. Insert shows no overt pathology in the CA3 hippocampal subfield. **c** Absence of Fluoro-Jade B-positive neurons in the hippocampus at 7 days post-bTBI. Insert shows no degenerating neurons in CA3. **d** and **e** Increased phosphorylated neurofilament immunostaining in CA3 at 3 days post-bTBI (**e**) compared to control subjects (**d**). **f** Bar graph of normalized signal of phospho-specific anti-neurofilament antibody, SMI-31, staining in hippocampal area CA3 from controls and rats at 3 days post-bTBI is shown. Signal was quantitated on *n* = 9–12 slides from 3 individual rats for each treatment group (3–4 slides/rat). Data are presented as mean ± SEM; *p < 0.05; Student’s *t-*test. **g** Swollen dystrophic axon in hippocampal CA1 stratum pyramidale at 3 days post-blast are indicated by arrowhead. **h** Axonal bulb in the hippocampal hilus at 7 days post-bTBI is indicated by arrowhead. Representative microscope pictures of hippocampal sub-regions immunostained with SMI-31 antibody (**d**, **e**, **g**, and **h**). Scale bars: 50 μm (**b**-**e**); 20 μm (**g** and **h**)

The hippocampus is critically involved in memory formation. Dysregulation of the hippocampal circuitry is thought to underlie some of the pathophysiological changes observed in TBI. Therefore, to better understand the impact of mild bTBI on neuronal circuitry associated with learning and memory, we specifically examined the neuropathological changes induced by mild bTBI in the hippocampus. Coronal hippocampal sections of rats subjected to bTBI exhibited no gross signs of neuropathology or tissue damage (Fig. 5a). H&E staining revealed no degenerating or ischemic neurons in the hippocampus following mild bTBI (Fig. 5b). Accordingly, FJB staining did not reveal signs of neurodegeneration in the hippocampus of bTBI-treated rats (Fig. 5c).

One of the primary neuropathological effects observed in most forms of TBI is diffuse axonal injury, which is characterized by distinct axonal pathology including swellings or varicosities along the length of axons, and the presence of axonal bulbs [26]. The axonal swellings contain accumulations of proteins, such as neurofilaments [27]. Previous studies reported that the phosphorylation level of neurofilaments is elevated following axonal injury and bTBI [28, 29]. Therefore, we examined the axonal integrity within the hippocampus from rats subjected to mild bTBI.

To evaluate the impact of mild bTBI on axonal integrity and phosphorylation levels of neurofilaments in the hippocampus, we performed immunohistochemical staining using the phospho-specific neurofilament antibody, SMI-31, followed by a semi-quantitative analysis. We found that immunoreactivity for phosphorylated neurofilaments was significantly increased in the hippocampal subfield CA3 of rats 3 days after exposure to mild bTBI as compared to controls (Fig. 5d-f). Interestingly, neurofilament phosphorylation has been implicated in neurofilament compaction [26], which may underlie axonal pathologies. Hence, we examined the integrity of the axonal architecture after mild bTBI. A detailed evaluation of axonal structures stained by SMI-31 revealed a small number of beaded or irregularly swollen dystrophic axons (Fig. 5g) as well as rare axonal bulbs in the hippocampus of rats exposed to mild bTBI (Fig. 5h). In controls no swollen dystrophic axons or axonal bulbs were observed (data not shown). These indicators of axonal injury observed in hippocampus of bTBI brains were accompanied by increased levels of reactive astrogliosis (Fig. 2g, h) and activated microglia (Fig. 3e, f). Together, these results show that mild blast forces induce neuropathological changes, including axonal injury and neuroinflammation in the hippocampus.

### Mild blast forces induce neurophysiological deficits in the hippocampus

**Fig. 6:**
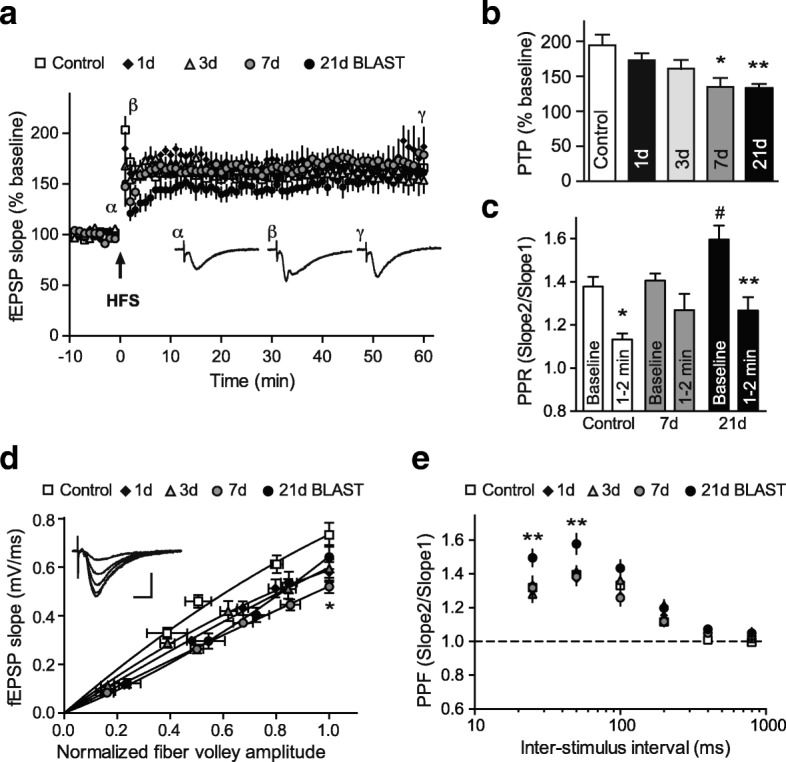
Mild bTBI caused deficits in basic synaptic properties and short-term plasticity. **a** Assessment of the effect of mild blast forces on long-term potentiation (LTP) in rats at 1, 3, 7 and 21 day(s) after bTBI exposure, as well as in controls. The graph shows the time-course of the field excitatory postsynaptic potential (fEPSP) slopes before and after high frequency stimulation (HSF) in percentage from the baseline. Insets show representative traces of recordings from control slices (α: baseline, β: post-tetanic potentiation (PTP) phase, γ: LTP phase). Arrowhead indicates the time point of HFS. **b** Summary of PTP changes in response to HFS shows a significant reduction at 7 and 21 days post-bTBI (*p < 0.05, **p < 0.01, vs. control, one-way ANOVA, Newman-Keuls post hoc). **c** Paired pulse ratio (PPR) at baseline and during PTP phase in slices from rats at 7 and 21 days post-bTBI, as well as in controls (*p < 0.05, **p < 0.01, vs. baseline, Wilcoxon test, ^#^*p* < 0.05, vs. control, Mann-Whitney test). **d** Input-output curves from fEPSP slopes against normalized fiber volley amplitudes. Connecting lines show a non-linear regression using a polynomial quadratic function for each group. Inset shows representative traces of recordings from control slices at different stimulation intensities. **e** Paired pulse facilitation (PPF) at different inter-stimulus intervals shows a significant difference at 21 days post-bTBI (**p < 0.01, two-way ANOVA, Tukey’s post hoc). All data are presented as mean ± SEM; *n* = 7–9 slices from 2 to 4 individual rats

Considering the importance of the hippocampus for higher cognitive functions, the effects of the blast-induced neuropathological changes (see above; Fig. 5) on neurophysiological outcomes were interrogated in the hippocampus. For this purpose, we investigated the effects of mild bTBI on basic synaptic properties and synaptic plasticity in acutely prepared rat hippocampal slices. High frequency stimulation (HFS) induced robust LTP at Schaffer collateral-CA1 synapses in bTBI rats, as well as controls (Fig. 6a). In control slices, tetanic stimulation resulted in a 166 ± 11% potentiation of fEPSP slope compared to baseline at 60 min post-stimulus (*p < 0.05, Student’s *t-*test). At 1, 3, 7 and 21 day(s) post-bTBI HFS induced comparable levels of LTP after 60 min (Fig. 6a), suggesting that bTBI-induced neuropathological changes did not affect LTP expression. In contrast, assessment of short-term synaptic responses revealed that post-tetanic potentiation (PTP) was significantly attenuated in slices from rats at 7 and 21 days post-bTBI (Fig. 6a, b; *p < 0.05, **p < 0.01, one-way ANOVA, Newman-Keuls post hoc). In order to explore a possible pre-synaptic effect, we employed the paired pulse facilitation paradigm to assess changes on neurotransmitter release. A significant reduction on paired pulse ratio (PPR) was observed in response to HFS in control slices (Fig. 6c; 1.38 ± 0.03 baseline for control to 1.14 ± 0.02 during PTP phase for control; *p < 0.05, *n* = 8, Wilcoxon test) indicating an increase in the probability of neurotransmitter release during the PTP phase. In contrast, PPR was attenuated during PTP in slices from rats at 7 days post-bTBI as compared to controls (Fig. 6c; 1.39 ± 0.03 baseline for 7d to 1.27 ± 0.07 PTP for 7d; *p* > 0.05, *n* = 8, Wilcoxon test). Interestingly, slices from rats at 21 days post-bTBI showed a significant increase in PPR at baseline compared to control (Fig. 6c; 1.38 ± 0.03 baseline for control to 1.60 ± 0.06 baseline for 21d; ^#^*p* < 0.05, *n* = 8, Mann-Whitney test), indicating changes in basal synaptic properties at 21 days post-bTBI that are likely to affect physiological neuronal functions. In conjunction with an increased PPR at baseline at 21 days post-bTBI, the PPR in response to HFS was significantly reduced during the PTP phase (Fig. 6c; 1.60 ± 0.06 baseline for 21d to 1.27 ± 0.06 PTP for 21d; **p < 0.01, *n* = 8, Wilcoxon test).

To assess hippocampal synaptic excitability, field potential recordings were used to derive input-output curves (Fig. 6d). The input-output curve for rats at 7 days post-bTBI was significantly reduced (Fig. 6d; *p* < 0.01, non-linear regression using a polynomial quadratic function). Consistently, the maximal response for rats at 7 days post-bTBI was significantly decreased (Fig. 6d; *p < 0.03, maximal stimulation in control vs. 7 day post-bTBI Mann-Whitney test). To further characterize the effect of bTBI on synaptic responses, paired pulse facilitation (PPF) was assessed at different inter-stimulus intervals (Fig. 6e). Slices from rats at 21 days post-bTBI showed significant increase in PPF at shorter inter-stimulus intervals (Fig. 6e; 20 and 50 ms, **p < 0.01, two-way ANOVA, Tukey’s post hoc). No difference in PPF was observed in slices from rats at 1, 3 and 7 day(s) post-bTBI as compared to controls.

Together, these results show that mild blast forces induced deficits in hippocampal circuitry basal synaptic properties and short-term plasticity, but did not alter LTP expression. These neurophysiological deficits were not detected prior to 7 days post-bTBI and were more pronounced by 21 days post-bTBI, suggesting that neuronal functions can be critically affected by mild bTBI even after 21 days. Interestingly, the primary injuries, such as microvascular damage and increased phosphorylation levels of neurofilament, as well as neuroinflammation had receded by 21 days post-bTBI, when neurophysiological deficits became most apparent.

### Mild blast forces induce biochemical changes in the hippocampus

TBI causes neuronal depolarization resulting in a large influx of ions. In response to activation of voltage-gated Ca^2+^ channels, high levels of glutamate are released, triggering excitotoxicity. Swelling of neurons, oxidative stress, and free radical production, all affect neuronal viability, contribute to neuronal death, and are associated with TBI pathology [30]. Following initial trauma, a delayed and spreading process of injury occurs. At the subcellular level, mitochondrial dysfunction and disruption in Ca^2+^-homeostasis have been implicated in TBI pathogenesis [31]. Over-activation of Ca^2+^-dependent enzymes such as the protease calpain may contribute to the etiology of TBI [32, 33].

**Fig. 7:**
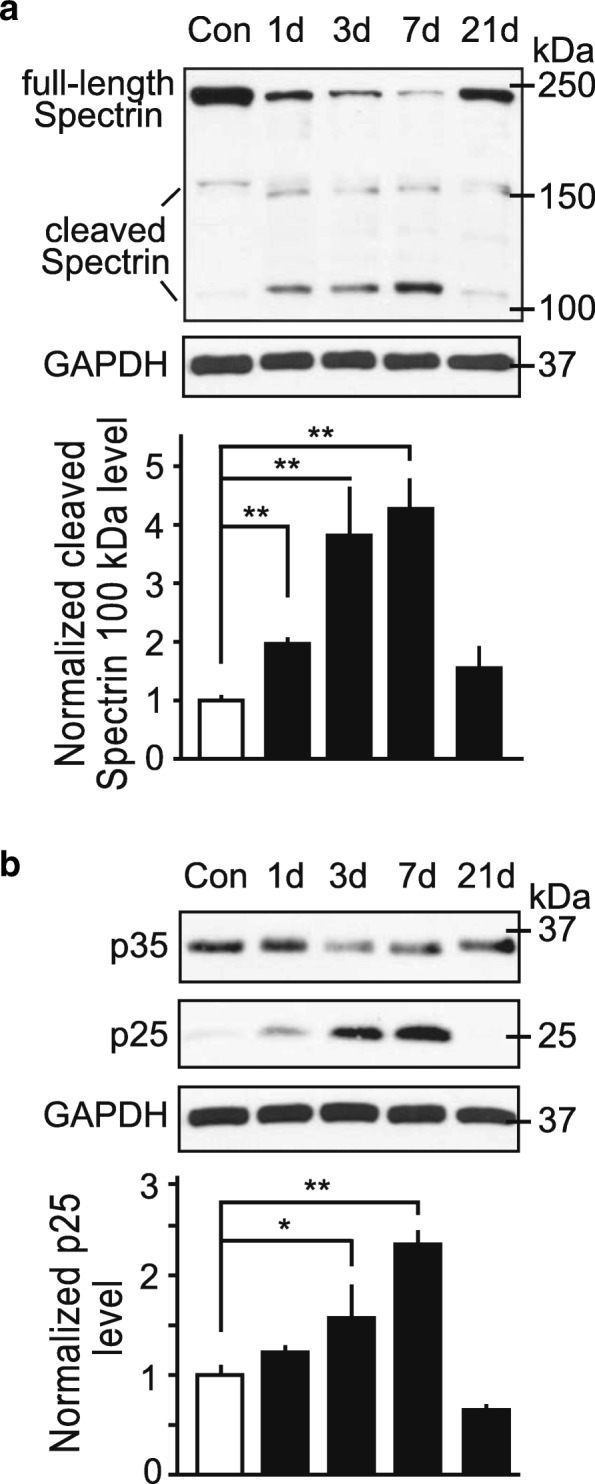
Mild bTBI induced proteolytic mechanisms associated with neuronal injury. **a** and **b** Representative immunoblot images (top) and quantitative analyses of immunoblot signals (bottom) are shown. Immunoblots of hippocampal lysates from control and blast-exposed rats at the indicated post-bTBI time points were probed for spectrin and its calpain-cleaved isoforms (**a**), as well as the Cdk5 activator p35 and its calpain-cleaved p25 fragment (**b**). All data are presented as mean ± SEM; *n* = 4–6/number of animals; *p < 0.05, **p < 0.01; one-way ANOVA with Bonferroni post hoc

Here, we examined the effects of mild bTBI on calpain activity in hippocampal lysates from blast-exposed rat brains using quantitative immunoblotting. As a marker of calpain activity, the levels of cleaved spectrin were assessed (Fig. 7a). The levels of the 100 kDa fragment of cleaved spectrin were significantly elevated at 1, 3 and 7 day(s) post-bTBI in comparison to controls. Another well-characterized calpain substrate is p35, the activating cofactor of the protein kinase Cdk5. Calpain-dependent cleavage of p35 produces the truncated protein p25. The resulting Cdk5/25 holoenzyme engenders aberrant activity, has been implicated in experimental TBI [14] and can contribute to neuronal cell death [34]. Consistent with an increase in calpain activity, p25 levels were significantly elevated at 3 and 7 days following mild bTBI (Fig. 7b). Taken together, these results demonstrate that effectors of excitotoxicity are invoked by mild bTBI, and may contribute to the etiology of TBI.

## Discussion

Growing evidence suggests that brain injury from blast exposure is a unique and particularly problematic form of neuropathology that may be linked to severe mental illness and chronic neurodegeneration [24]. Here, we investigated the pathophysiological effects induced by mild blast forces in adult male rats at various time points after bTBI. Consistent with our previous studies [6], the overpressure wave generated by the scaled, bench-top blast tube used here did not cause macroscopic neuropathological changes, such as tissue damage, hemorrhage, hematoma, or contusion to the brain or dura. However, close examination revealed that one single mild overpressure wave induces microvasculature damage and axonal injury accompanied by neuroinflammation in various brain regions including cortex, striatum and hippocampus. These neuropathological changes are correlated with deficits in neurophysiological outcomes, including basic synaptic properties and short-term plasticity in hippocampus. Finally, a biochemical analysis revealed that the Ca^2+^-dependent protease calpain is overactivated after mild bTBI, indicating that Ca^2+^ homeostasis is disturbed by mild blast forces. Together, these results show that even mild blast forces can cause subtle, but deleterious pathophysiological changes in the absence of major neuropathological injuries.

Numerous studies have demonstrated that basic synaptic properties and synaptic plasticity are affected in rodent models of TBI, including fluid percussion injury (FPI) and controlled cortical impact (CCI) [35–39]. The effect of primary bTBI on neurophysiological outcomes is less explored. One study reported that CA1 LTP was reduced in mouse brains at 2- and 4-weeks after a single sub-lethal blast (167 kPa/msec) [4]. Another study found that low-level primary blast trauma is associated with electrophysiological white matter dysfunction at 2 weeks post-injury [40]. Our results show that mild blast forces induce deficits in basal synaptic properties and short-term plasticity, but do not alter LTP expression. These neurophysiological deficits are not observed prior to 7 days post-bTBI and are more pronounced by 21 days post-bTBI, suggesting that neuronal functions can be critically affected by some process set in motion by mild bTBI. Furthermore, these findings also indicate that primary injuries, such as microvascular damage and neuroinflammation, as well as increased phosphorylation levels of neurofilaments do not directly induce neurophysiological deficits, as the primary injuries are present early on following bTBI but had receded by 21 days post-bTBI, whereas the neurophysiological deficits appear only after 7 days post-bTBI. Thus, our data suggests that mild blast forces can induce long-lasting pathophysiological changes, other than the primary injuries, that lead to deficits in basal synaptic properties and short-term plasticity weeks after the initial injury that may impact brain functions.

Despite the absence of gross neuropathological effects, such as tissue damage, hemorrhage or contusion, we find increased astrogliosis and microglia activation after mild bTBI in cortex, hippocampus and striatum. Consistent with previous TBI studies [41], we observed neuroinflammation during the initial phases of injury, namely at 1, 3 and 7 day(s) after bTBI. By 21 days post-bTBI astrogliosis and microgliosis levels were comparable to controls showing that the acute early activation of gliosis is significant for injury expansion but resolves over time. Moreover, previous research using different blast intensities in rats and organotypic hippocampal slice cultures showed that increasing blast intensity resulted in elevated levels of astrogliosis and microglia activation [6, 42].

Our results show that the Ca^2+^-dependent protease calpain is overactivated post-bTBI as indicated by increased degradation of its substrates spectrin and the Cdk5 activator p35 (Fig. 7). Compromised microvascular integrity is often related to brain injuries involving excitotoxicity. Activation of Ca^2+^-dependent proteases, such as calpain, is a predicted outcome of membrane depolarization and loss of Ca^2+^ homeostasis. Spectrin is involved in actin binding and maintaining the shape of synapses thereby regulating synaptic functions, including synaptic plasticity [43]. Thus, calpain activation and cleavage of spectrin is consistent with deficits in axonal architecture and disruption of synaptic plasticity [44]. Indeed, rats exposed to bTBI can exhibit shortened axon initial segments, suggesting such subcellular changes [45]. Moreover, spectrin cleavage in the corpus callosum after bTBI has been suggested to attenuate overall electrophysiological responses [40] and neuronal death [46]. Calpain-dependent conversion of p35 to p25 results in dysregulation of the protein kinase Cdk5, causing relocation of the protein kinase and redirection towards aberrant substrates that mediate neuronal injury [34]. Thus, exposure to moderate blast forces may initiate subtle but still meaningful neuropathological processes.

Taken together, the results presented here demonstrate that even mild blast forces can induce a panoply of pathophysiological effects with long-lasting consequences for neuronal functions. Future studies into the molecular and cellular changes underlying these pathophysiological changes will be needed to advance our understanding of bTBI etiology. Finally, the changes observed in this study, would probably not be detected using structural brain imaging techniques, giving a rational as to why most structural imaging studies of mild TBI in human failed to reveal significant insights. However, our results suggest that even mild bTBI might have significant neuronal/functional consequences that occur long after the actual blast exposure/injury.

## Abbreviations

ACSF: Artificial cerebrospinal fluid
bTBI: Blast-induced traumatic brain injury
CCI: Controlled cortical impact
Cdk5: Cyclin-dependent kinase 5
fEPSP: Field excitatory post synaptic potential
FJB: Fluoro-Jade B
FPI: Fluid percussion injury
GFAP: Glial fibrillary acidic protein
H&E: Hematoxylin and eosin
HFS: High frequency stimulation
IACUC: Institutional Animal Care and Use Committees
Iba1: Ionized Ca^2+^-binding adapter molecule
IgG: Immunoglobulin G
LTP: Long-term potentiation
PPF: Paired pulse facilitation
PPR: Paired pulse ratio
PTP: Post-tetanic potentiation
RRID: Research Resource Identifiers
TBI: Traumatic brain injury
