# Correction to: Exposure to mild blast forces induces neuropathological effects, neurophysiological deficits and biochemical changes

**DOI:** 10.1186/s13041-021-00872-w

**Published:** 2021-11-10

**Authors:** Adan Hernandez, Chunfeng Tan, Florian Plattner, Aric F. Logsdon, Karine Pozo, Mohammad A. Yousuf, Tanvir Singh, Ryan C. Turner, Brandon P. Lucke-Wold, Jason D. Huber, Charles L. Rosen, James A. Bibb

**Affiliations:** 1 Department of Psychiatry, University of Texas Southwestern Medical Center, Dallas, TX 75390 USA; 2 Center for Translational Neurodegeneration Research, University of Texas Southwestern Medical Center, Dallas, TX 75390 USA; 3 Department of Neurosurgery, West Virginia University School of Medicine, Morgantown, WV 26506-9183 USA; 4 Department of Basic Pharmaceutical Sciences, West Virginia University School of Medicine, Morgantown, WV 26506-9530 USA; 5 Departments of Surgery, Neurobiology, and Neurology, The University of Alabama at Birmingham Medical Center, 1720 2nd Ave S, THT 1052, Birmingham, AL 35294 USA

## Correction to: Mol Brain (2018) 11:64 10.1186/s13041-018-0408-1

Following publication of the original article [1], the authors identified an error in the author name of Brandon P. Lucke-Wold.

The incorrect author name is: Brandon P. Luke-Wold

The correct author name is: Brandon P. Lucke-Wold

The author group has been updated above and the original article [1] has been corrected.
